# In Situ Bioprinting Enhances Bone Regeneration in a Live Animal Model with Craniofacial Defect

**DOI:** 10.1021/acsbiomaterials.5c00780

**Published:** 2025-07-24

**Authors:** Osama Ali Hindi, Begum Pinarbasi, Merve Bakici, Oya Burcin Demirtas, Seyda Gokyer, Arda Buyuksungur, Kaan Orhan, Cagdas Oto, Pinar Yilgor

**Affiliations:** 1 Department of Biomedical Engineering, 37504 Ankara University, Ankara 06100, Turkiye; 2 Department of Surgery, Kirikkale University Faculty of Veterinary Medicine Kirikkale 71450, Turkiye; 3 Department of Pathology, Ankara University Faculty of Veterinary Medicine Ankara 06100, Turkiye; 4 Department of Basic Medical Sciences, Ankara University Faculty of Dentistry, Ankara 06100, Turkiye; 5 Department of Dentomaxillofacial Radiology, Ankara University Faculty of Dentistry, Ankara 06100, Turkiye; 6 Ankara University Medical Design Research and Application Center MEDITAM, Ankara 06100, Turkiye; 7 Department of Anatomy, Ankara University Faculty of Veterinary Medicine Ankara 06100, Turkiye

**Keywords:** in situ bioprinting, bioink, live animal model, craniofacial bone regeneration, 3D bioprinting

## Abstract

In situ bioprinting represents an innovative approach in tissue engineering and regenerative medicine, enabling direct deposition of bioinks within the body to create or repair tissues at the target site. This technique leverages advanced bioprinting technologies to deliver cells, biomaterials, and bioactive molecules in a precise, controlled manner, offering the potential for on-demand tissue repair and minimizing the need for extensive surgical intervention. In this research, we apply for the first time in the literature a standard 3D bioprinter to perform in situ bioprinting over the bone defects of live animals under anesthesia and discuss the bone regeneration potential. For this, critical-sized bone defects were created on the parietal bones of the rabbits, followed by the application of autologous adipose-derived stem cell-laden bioink using a 3D bioprinter. Postoperative evaluations included micro-CT and histopathological analysis to assess bone healing and bone-material integration. The results demonstrated successful bone regeneration with the in situ bioprinting approach, as compared to the sham and the use of bioink-only. In conclusion, this study contributes to the growing body of evidence supporting in situ 3D bioprinting as a viable and promising technique for craniofacial bone regeneration, with potential implications for broader clinical relevance and paves the way for future clinical applications.

## Graphical abstract

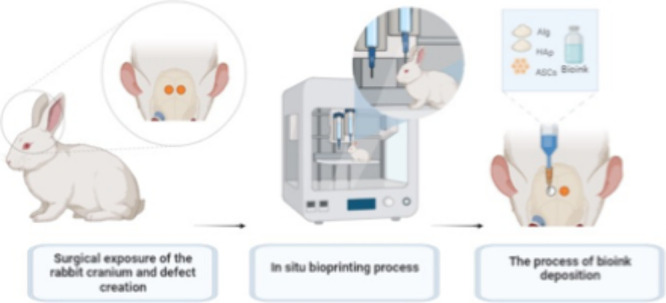

## 1 Introduction

In situ bioprinting of bone represents a transformative approach in the field of regenerative medicine, combining principles of 3D bioprinting with surgical methodologies to directly repair and regenerate bone tissues at the injury site. This technique enables the precise deposition of bioinks that contain osteoinductive and osteoconductive materials, living cells, and bioactive agents, specifically engineered to promote regeneration at the defect site.^1–3^ Unlike traditional bone grafting methods, in situ bioprinting offers the advantage of real-time customization, enabling tailored scaffolds that conform to the unique geometry of each defect and integrate seamlessly with surrounding native tissues.^4–6^

Recent advancements in hand-held bioprinting devices have showcased an innovative approach for in situ applications where precision is less critical than adaptability. Unlike standard 3D bioprinters, hand-held bioprinters allow surgeons to maneuver directly over the defect site, applying bioinks to fit irregular shapes and complex contours. For instance, Di Bella et al.^7^ demonstrated that hand-held bioprinting could facilitate cartilage regeneration, with promising results in rapidly filling joint defects during surgical procedures, Duarte Campos et al.^8^ applied hand-held bioprinting for bone scaffolding directly at defect sites, achieving satisfactory tissue integration and structural stability without the need for complex setup. Even though these devices offer flexibility and ease of use, their manual application lacks the precision achievable with stationary, computer-controlled 3D printers. Consequently, while hand-held bioprinters can accommodate irregular defect shapes, their output may vary due to manual control, making them more suitable for situations where exact spatial arrangement and complex structures are not as essential.

Alternatively, integrating complex robotic arms into in situ bioprinting offers enhanced precision, but these systems present significant practical challenges. Robotic devices require extensive calibration, advanced imaging-guided mapping, and skilled operators, often making them impractical for routine clinical settings.^9–11^ These robotic solutions tend to be bulky and costly, limiting their applicability in a point-of-care environment. In contrast, modified conventional 3D printers designed for in situ use, present a promising compromise between precision and practicality. They can produce reproducible, accurately layered constructs while being more user-friendly and cost-effective than robotic counterparts. For example, Pati et al.^12^ explored the use of traditional 3D bioprinting frameworks to generate tissue analogs, which could be applied to bone and cartilage regeneration directly on-site with minimal setup. These systems also integrate into surgical workflows, allowing clinicians to deploy customized constructs tailored to specific defect morphologies, thus advancing the potential for broader application of in situ bioprinting in clinical practice.

The development of bioinks suitable for bone tissue engineering has been central to advancing in situ bioprinting techniques. These bioinks must mimic the mechanical and biological properties of native bone, including stiffness, porosity, and bioactivity, while also supporting cell survival, proliferation, and differentiation.^13–16^ Various materials, including calcium phosphate ceramics, hydrogels, and polymer-based composites, have been explored for their potential to facilitate osteogenesis and improve the mechanical strength of bioprinted constructs.^17–19^

The use of in situ bioprinting is particularly advantageous in complex or irregularly shaped bone defects, which are difficult to treat using conventional methods.^9,12,20^ Advanced imaging technologies, such as computed tomography (CT), magnetic resonance imaging (MRI) and surface scanning^21^ enable the precise mapping of bone defects, which in turn informs the bioprinting process, ensuring that the bioprinted scaffold matches the defect geometry accurately.^7,22,23^ Furthermore, in situ bioprinting minimizes the risk of contamination and reduces surgical time, making it a promising approach for point-of-care applications in orthopedic surgery.^8,24,25^

Recent studies in cranial bone tissue engineering have increasingly focused on the development of bioinspired, 3D-printed scaffolds that closely mimic the structural, mechanical, and biological properties of native bone. A comprehensive review by Khorasani and Vahidi^26^ outlines the evolution of scaffold design, material selection, and computational optimization techniques over the past decade, emphasizing the potential of patient-specific constructs in enhancing regeneration outcomes. Building on this foundation, bionic mineralized scaffolds capable of enzymatically induced in situ mineralization, and prevascularized bone organoid system using 3D bioprinting and bioactive hydrogels to promote rapid, vascularized bone regeneration through synergistic signaling pathways were developed.^27,28^ Furthermore, the efficacy of dynamic culturing in PCL/GelMA composite scaffolds, resulting in enhanced vascular infiltration and accelerated healing in critical-sized cranial defects were investigated.^29^

The novelty of this research lied in the direct application of bioprinting onto live animals under anesthesia, which enhances the clinical relevance of the findings and paves the way for future clinical applications. Three experimental groups were established: in situ bioprinting of the cell-laden bioink, in situ bioprinting of the osteoinductive hydrogel, and the sham. The alginate-hydroxyapatite bioink was optimized for printing directly onto the defect sites, and the bone regeneration process was monitored through micro-CT scanning and histopathological analysis. Results showed that the cell-laden bioink exhibited superior bone regeneration, with increased bone formation and integration compared to the control groups. The findings highlight the potential of in situ bioprinting for enhancing craniofacial bone repair, demonstrating significant improvements in both bone quality and healing time. The schematic representation of the study is provided in Figure 1.

**Figure 1:**
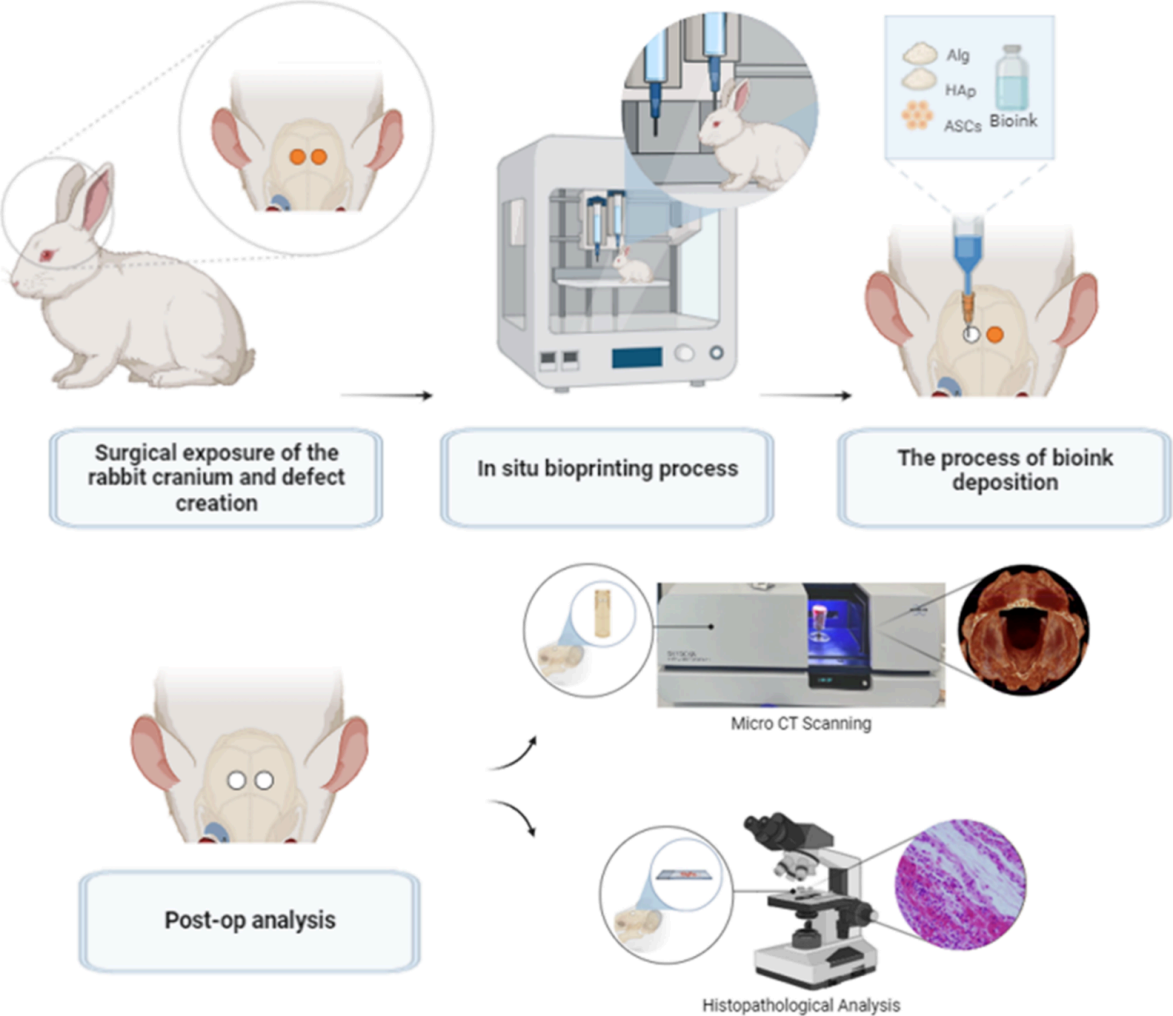
Schematic representation of the in situ bioprinting process on the live rabbit craniofacial bone under anesthesia, and the following postop analysis including micro-CT and histopathology.

## 2 Methods

### 2.1 Bioink

#### 2.1.1 Adipose-Derived Stem/Stromal Cell Isolation and Culture

Adipose-derived stem cells (ASCs) were isolated from subcutaneous fat obtained from rabbits as described before,^30^ following a protocol approved by Ankara University Animal Research Ethics Committee (approval number 2022–3–26). Briefly, abdominal fat pad was digested with 0.1% collagenase type 1, centrifuged, and the stromal vascular fraction was cultured to obtain adherent cells (P0). After cryopreservation, P1 ASCs were thawed in DMEM with 10% FBS, 1% P/S, and 1 ng/mL FGF-2, and used without in vitro culture to simulate the clinical conditions for in situ bioprinting, where isolation and concurrent use of autologous ASCs is aimed.^31^

#### 2.1.2 Bioink Preparation and Printability Analysis

A combination of alginate (Sigma-Aldrich, medium viscosity) and hydroxyapatite (HAp) (Merck) was tested to optimize the printability and the mechanical properties of the bioink. Various HAp concentrations (4–24 wt % w/v) was mixed with the alginate solution (6%, w/v) to prepare the osteogenic bioink.

Optimization of the bioprinting parameters for the bioink was carried out using a linear filament model for varied pressure values (1.5–3.5 bar) and HAp concentrations (4–24% w/v), and the bioprinted constructs were visualized by fluorescence microscopy (Zeiss Axio Observer). The filament thicknesses were measured using ImageJ Software (NIH).

For in situ bioprinting on live rabbits, a sterile bioink of 6% (w/v) alginate and 8% (w/v) HAp was prepared and mixed with 5 × 10^6^ ASCs/mL. For this, the powders (alginate and HAp) were UV sterilized for 30 min, then mixed with 1 mL of sterile PBS and 10 μL of penicillin/streptomycin (P/S). ASCs were introduced within the bioink immediately before bioprinting. A sterile 0.5 M calcium chloride solution was used to stabilize the bioprints.

#### 2.1.3 Rheological Characterization of Bioinks

Rheological analysis of alginate and alginate-hydroxyapatite (Alg/HAp) composite hydrogels was performed using a on a Malvern Kinexus rheometer with a 25 mm plate diameter and a 500 μm gap between the plate and sample to evaluate their viscoelastic properties. Frequency sweep and amplitude sweep tests were conducted on both non-cross-linked and ionically cross-linked hydrogels containing varying HAp concentrations (4%, 8%, 16%, and 24% w/v). Frequency sweeps were performed at a constant strain within the linear viscoelastic region to determine the storage modulus (G′) and loss modulus (G″) across a range of angular frequencies. Amplitude sweeps were conducted to assess the response of the material to increasing strain and to define the limit of linear viscoelasticity.

#### 2.1.4 Mechanical Testing

The mechanical properties of 3D printed alginate and alginate/HAp scaffolds were evaluated using a Universal Testing Machine (Shimadzu AGS-X, Japan). Samples (n = 3, 8 × 8 x 5 mm) were subjected to uniaxial compression with a 50 N load cell at 1 mm/min. YounG′s modulus (YM) was calculated from the stress–strain curve in the elastic region, and ultimate compressive strength (UCS) was determined as the maximum stress before failure.

### 2.2 Precision 3D Scanning and Digital Modeling

The craniofacial region of the rabbit was scanned from three different angles to ensure that all surfaces of the craniofacial bone are captured by the camera, using a high-resolution 3D scanner (Artec Eva, LU) to obtain precise digital models of the defect site. These scans were used to generate accurate 3D models, which allowed for customized surgical planning and alignment during the in situ bioprinting process (Figure 2A). Subsequently, the scanned data was restored to STL format with reverse engineering software (Fusion 360) enabling precise control of bioink deposition in subsequent experiments.

**Figure 2:**
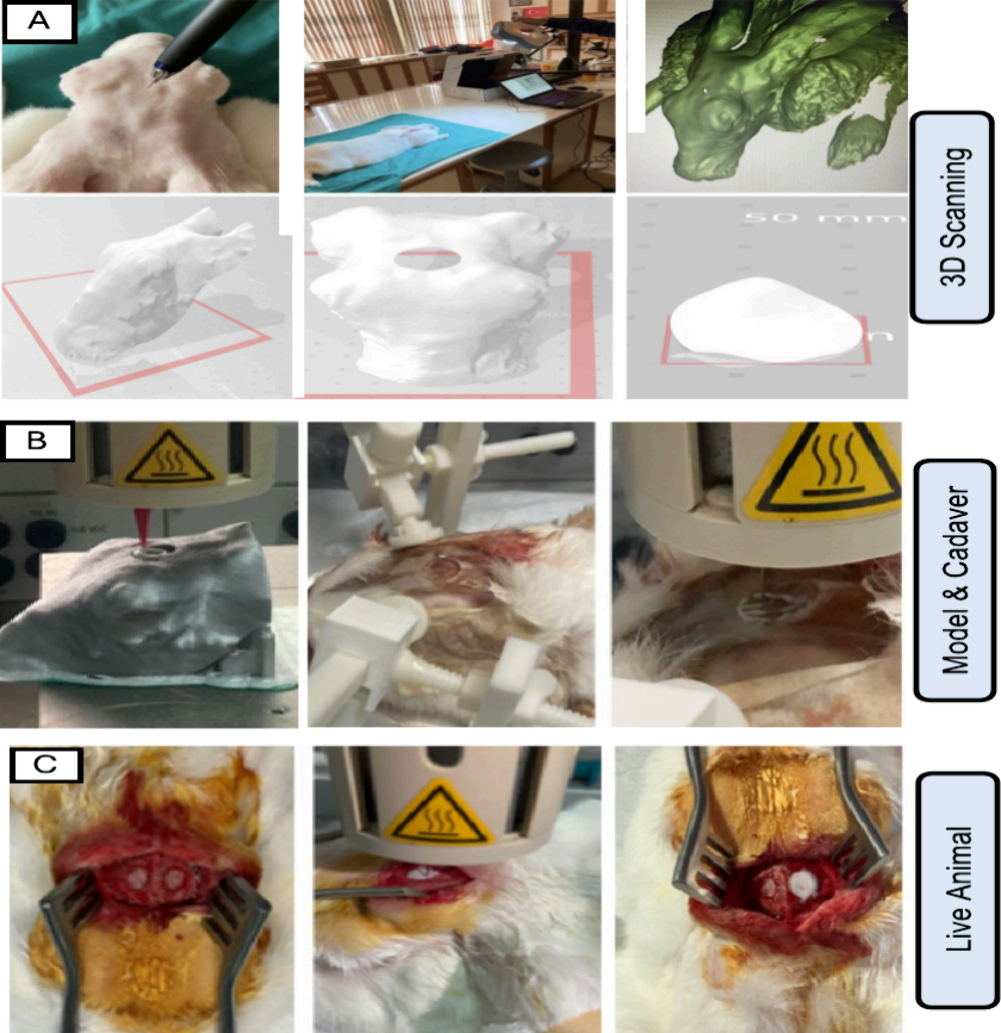
(A) Workflow of precision 3D scanning and digital modeling of the rabbit craniofacial bone. (B) Optimization of the bioprinting procedure on the 3D-printed rabbit cranium model, and the rabbit cadaver. (C) In situ 3D bioprinting on live animal.

### 2.3 Optimization of in situ 3D Bioprinting Procedure on Rabbit Cranium Model and Cadaver

Initial experiments for in situ bioprinting were conducted on rabbit cranium models and rabbit cadavers to fine-tune the bioink extrusion process (Figure 2B). These tests allowed for adjustments to the bioprinting workflow without the influence of live tissue responses. Parameters such as HAp concentration within alginate matrix (4–24% w/v) and print pressure (1–3 bar) were optimized to achieve reproducible bioprinted structures that fill the defects precisely according to the 3D model created from surface scanning of the defect. 3D Bioplotter (EnvisionTec, Germany) device was used to 3D print the bioink under sterile conditions with a 23G nozzle tip.

### 2.4 In Situ Bioprinting on Live Animals

All animal procedures were performed following a protocol approved by Ankara University Animal Research Ethics Committee (approval number 2022–3–26). Healthy, male New Zealand white rabbits, 6 months of age with an average weight of 3.5 kg were used for the study. All rabbits were singly housed in standard stainless-steel cages, with ad libitum water and a standard commercial rabbit diet under the conditions of 55% humidity, room temperature (20–24 °C), a 12-h light cycle, and a 12-h dark cycle. Rabbits were divided into three groups (cell-laden bioink, acellular bioink, sham) with two defects created on each animal. Six defects were created for each experimental group.

All interventional processes were carried out under sterile conditions and general anesthesia. Induction of general anesthesia was achieved by intramuscular administration of ketamine (35 mg/kg) in combination with xylazine (10 mg/kg). Maintenance of anesthesia was provided by total intravenous anesthesia (TIVA) with propofol at 0.5 mg/kg/min. The rabbits were intubated with a 3.0 mm cuffed endotracheal tube, and a purple-colored IV catheter was placed in the marginal vein of the ear. A balanced electrolyte solution was administered at 5 mL/kg/h during anesthesia. After clipping the skull hair and cleaning the area with alcohol and iodine solutions, eye lubricant was applied to both eyes. A preoperative antibiotic, ceftriaxone at 50 mg/kg, was administered intramuscularly.

A 4 cm skin incision was made along the sagittal line on the top portion of the skull, and the periosteum was carefully faltered to expose the calvarium. Critical-sized circular defects with a diameter of 8 mm were created bilaterally on the parietal bone using a hand motor and round-tipped burr with continuous saline irrigation.

In situ bioprinting was performed on live rabbits under anesthesia over these surgically created bone defects. The bioinks, both with and without cells, were 3D printed directly into the defect using the 3D Bioplotter (EnvisionTec, Germany) (Figure 2C).

After the bioprinting process, the periosteum, subcutaneous tissue, and incised skin were closed with a monofilament absorbable suture material (4/0 Polyglycolic acid) layer by layer in a simple continuous and simple interrupted pattern, respectively.

Routine postoperative care followed surgery. Meloxicam (1 mg/kg) and ceftriaxone (50 mg/kg) were administered intramuscularly and continued for 72 h postop. The animals were evaluated daily for wound healing progress, signs of pain or distress, and general activity.

During the first, second-, and third-weeks postsurgery, animals underwent computed tomography (CT) scanning to assess bone healing. At the conclusion of the sixth postoperative week, euthanasia was performed using an overdose of intravenous sodium pentothal. Histopathological examination, and microcomputed tomography (micro-CT) were conducted on the operated bone areas.

### 2.5 Post-Op Analysis

#### 2.5.1 Micro-CT Scanning

A high-resolution, desktop Micro-CT system (Bruker Skyscan 1275, Belgium) was used to scan the specimens. The scanning conditions were: 80 kVp, 125 mA, with 1 mm Al filter, 25 μm pixel size. To minimize artifacts, flat field correction of the detector was carried out. Each sample was scanned with 360° through rotation at 0.2 step. The mean time of scanning was around 30 min.

The NRecon software (ver. 1.7.4.2, Bruker Skyscan, Belgium) was used to obtain axial, two- dimensional (2D) images. The reconstruction parameters were used as ring artifact correction was set to 7, smoothing was 3 and the beam artifact correction was set at 38%. Image conversion limits were used as 0.0–0.04 for all the samples. NRecon the images obtained by the scanner were reconstructed to show 2D slices of the specimen. CTAn (ver. 1.23.0.2+, Bruker Skyscan, Belgium) was used for the quantitative 3D and 2D measurements of the samples.

After reconstruction, region of interest (ROI) was drawn to include the 3D printed material within the sample using CTAn software, and specifications of the program was used to analyze the 3D microarchitecture of newly formed bone tissue. To analyze the newly formed tissues, global thresholding was used for all the samples and the limits of the threshold were 50–255 (in gray scale values), the upper limit was at the top end of the brightness spectrum representing the highest bone density value. After thresholding (binarization) process, an imposed image of black/white pixels was achieved. Then, separately for each slice, a region of interest was chosen to contain 3D printed material to allow calculation of new bone tissue. Also, for the determination of the Bone Mineral Density (BMD), HAp calibration blocks with density of 0.25 g/cm^3^ and 0.75 g/cm^3^ was used. The calibration curve was used to determine the BMD values of the samples.

CTVox (ver. 3.3.0 r1403, Bruker Skyscan, Belgium) was used for the visualization of the samples in 3D. All specifications of the program were used to analyze the 2D and 3D microarchitecture of each sample. The following structural parameters were measured; percent object volume, object surface/volume ratio, object surface density, structure model index, structure thickness, structure linear density, structure separation, connectivity, connectivity density. Those parameters were calculated in 3D based on the volume of the ROI.

#### 2.5.2 Histopathological Analysis

Samples from 8 rabbits were collected and fixed for 48 h in neutral buffered formalin then decalcified in EDTA and hydrochloric acid solution (Biocal C, RRDC3-E, Atom Scientific LTD) for 3 days at 37 °C with daily solution replacement. They were dehydrated in a graded series of ethanol (70%–100%), cleared with xylene and subsequently embedded in paraffin. Three serial 5 μm sections were collected from each sample and stained with hematoxylin and eosin (H&E, Merck) and Masson’s trichrome (Beslab) for histopathological evaluation. Subsequently, the specimens were examined under light microscope (Olympus BX51TF, Japan) in a blinded manner.

#### 2.5.3 Immunofluorescence Staining

Sections were obtained as described above and incubated with phosphate-buffered saline (PBS) containing 0.1% Tween20 and 1% bovine serum albumin (BSA) for 2 h at 4 °C to prevent nonspecific binding and permeabilize cell membranes. Specimens were then incubated with primary antibodies for osteopontin (1:500) (antiosteopontin antibody, Abcam ab8448, mouse) and CD31 (1:1000) (anti-CD31 antibody, Abcam ab182981, rabbit) diluted in PBS containing 1% BSA overnight at 4 °C. Afterward, sections were incubated with secondary antibodies Alexa Fluor 488-conjugated goat antimouse IgG (1:200) and Alexa Fluor 555-conjugated goat antirabbit IgG (1:200) for 1 h at room temperature. Nuclei were counter labeled with DAPI and visualized on a confocal laser scanning microscope (Leica, Germany).

### 2.6 Statistical Analysis

All quantitative results were expressed as means ± standard deviation (*n* ≥ 3). Data was analyzed with statistically significant values defined as *p* < 0.05 based on one-way analysis of variance (ANOVA) followed by Tukey’s test for determination of the significance of difference between different groups (*p* ≤ 0.05).

## 3 Results

### 3.1 Printability Analysis on the Model, Cadaver, and the Live Animal

Alginate was used as the bioink material due to its biocompatibility, ease of gelation through ionic cross-linking, and widespread use in bioprinting applications. Moreover, HAp was incorporated within the alginate matrix to obtain an osteoconductive bioink. Figure 3A presents the printability of alginate-hydroxyapatite (Alg-HAp) bioinks at varying hydroxyapatite concentrations (4%, 8%, 16%, and 24%, w/v) under different extrusion pressures.^32^ All tested Alg-HAp formulations were nonprintable at lower pressures (≤1 bar). As extrusion pressure increased, printability was achieved across all HAp concentrations, with a threshold pressure of approximately 1.5 bar required for successful extrusion. Notably, despite increasing HAp content, the printability threshold remained relatively consistent, suggesting that HAp incorporation up to 24% did not significantly alter the extrusion pressure required for maintaining printable conditions. However, at higher hydroxyapatite concentrations (16% and 24%), nozzle clogging was frequently observed, leading to inconsistent extrusion and reduced printing reliability (Figure 3 **B)**. It was also observed that, presence of 4–6% HAp in the bioink results in thickening of the extruded filaments with increasing pressure, whereas its further increase did not significantly alter the bioprinted filament diameter (Figure 3C). Therefore, 8% HAp was selected as the optimal concentration, as it provided a balance between bioink printability and clogging issues. These findings highlight the importance of optimizing extrusion pressure and HAp content to ensure bioink printability while maintaining structural fidelity and desirable bioink properties. The printed structure during the biofabrication procedure is shown in Figure 3 **D.**

**Figure 3:**
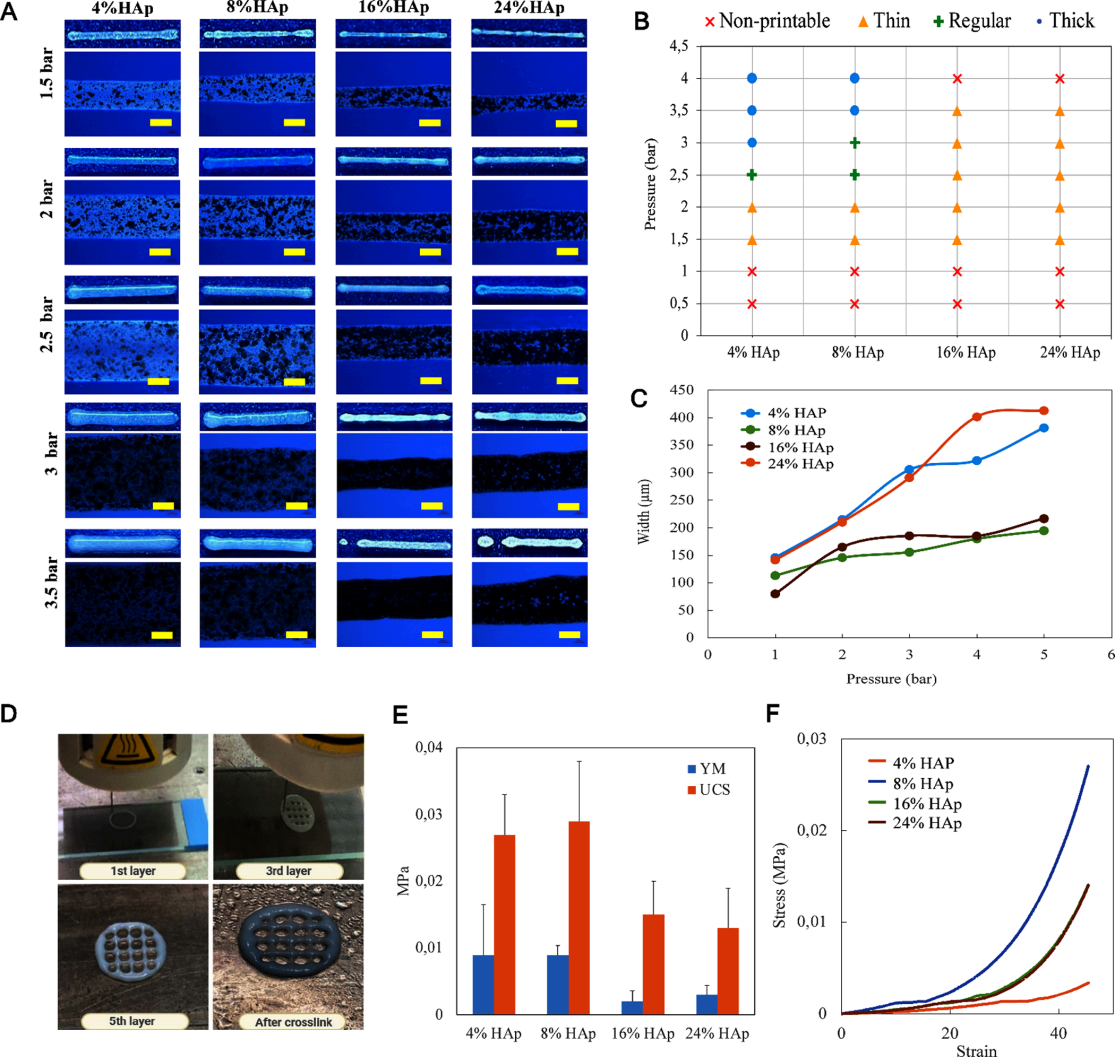
(A) Printability analysis images as line models of 6% (w/v) alginate and varying HAp concentrations (scale bars: 100 um). (B) Summary plot for the printability analysis of alginate/HAp bioink at varied pressures. (C) Width vs pressure (1–5 bar) graph for each HAp concentration at varied pressure values. (D) 3D-printed 6% Alg/8% HAp scaffolds at 10 × 10 × 5 mm dimensions. (E) Mechanical test results of alginate/HAp scaffolds. YM: YounG′s modulus, UCS: Ultimate compressive strength, and (F) Typical stress–strain curves of the samples.

Figure 3E presents the YounG′s modulus and ultimate compressive strength of alginate-hydroxyapatite (Alg-HAp) composites with varying HAp concentrations. The highest YounG′s modulus values observed for 6% Alg/4% HAp and 6% Alg/8% HAp. However, a decline is noted as the HAp content increases beyond 8%. Similarly, the ultimate compressive strength reaches its peak at 6% Alg/8% HAp, followed by a decrease with further HAp additions. The reduction in mechanical properties at higher HAp concentrations (16% and 24%) may be attributed to phase separation, inadequate polymer-HAp interactions, or an increase in brittleness. These findings suggest an optimal HAp concentration around 8% for maximizing mechanical performance in the Alg-HAp composite system. The right-hand side graph in Figure 3F represents typical stress–stress curves for the alginate-HAp composite bioinks used.

Rheological analysis revealed that both non-cross-linked and ionically cross-linked Alg/HAp composite hydrogels exhibited dominant elastic behavior (G′ > G″) across all tested HAp concentrations (Figure S1). In frequency sweep tests, the storage modulus increased with HAp content, particularly in the cross-linked hydrogels, indicating enhanced stiffness and elastic character. The 8% HAp formulation, which was selected for in vivo application, demonstrated a favorable balance of mechanical strength and processability. Amplitude sweep tests confirmed that the hydrogels maintained linear viscoelastic behavior over a broad strain range, with higher HAp content generally increasing the limit of linear viscoelasticity. These findings support the mechanical stability and printability of the bioink during and after the extrusion process.

The printability analysis was conducted on the glass slides and moreover, the proper 3D printing in accordance with the defect model was also verified on the 3D printed rabbit skull models and rabbit cadaver (Figure 2B). These tests allowed for adjustments to the bioprinting strategy without the influence of live tissue responses, prior to the experiments with the live animals. As demonstrated in Figure 2, it was verified that bioprinting was achieved both on the models, the cadaver and the live tissue, despite the inherent challenges of working with live tissue. The scaffolds not only adhered well to the defect areas but also provided stability and conformed to the tissue contours effectively. Visual assessments revealed that the printed scaffolds maintained their shape and integrity throughout the procedure. Table 1 provides the quantitative data on the in situ bioprinting procedure applied.

**Table 1:** Quantitative Data on the In Situ 3D Bioprinted Samples on Live Rabbit Models

parameter	value (mean ± SD)
scaffold dimension	8 × 8 × 5 mm
layer thickness	0.33 ± 0.05 mm
coverage area	64 mm^2^
printing time	8 ± 0.5 min
coverage efficiency	100%

### 3.2 Micro-CT Scanning and Quantitative Analysis

The optimized bioink (6% Alg/8% HAp) and bioprinting parameters were used to in situ bioprint cell-free and cell-laden bioink within the craniofacial defects. Defects left empty (sham), and bioprinted with cell-free and cell-laden bioink were analyzed. Key parameters such as bone volume, bone surface density, and structural properties were measured using CT imaging to provide a comprehensive analysis of the procedure.

It was observed that the defects remain unbridged in the sham, where defect bridging were obtained in the in situ bioprinted defects, with cell-laden bioink providing enhanced bone regeneration and defect coverage (Figure 4A) as compared to the cell-free bioink. The average bone volume for cell-free defects was 27.2 ± 1.3 mm^3^, while the cell-laden group showed almost full coverage and substantial bone formation, achieving almost complete defect coverage and an average bone volume of 50.7 ± 3.1 mm^3^. Sham defect had a minimal volume of 5.3 ± 0.9 mm^3^, indicating that the in situ bioprinted Alg/HAp material significantly promotes bone regeneration.

**Figure 4:**
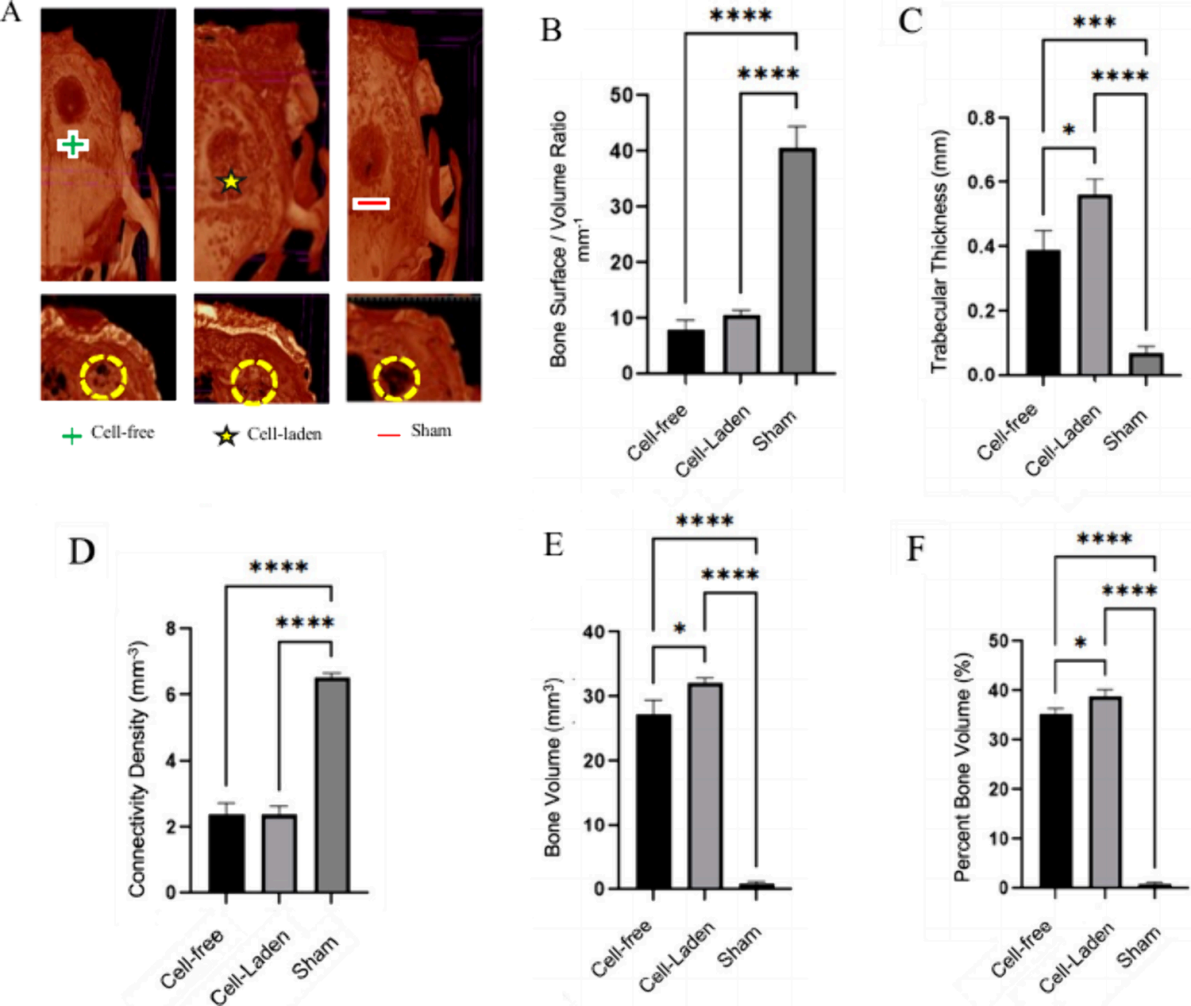
Micro-CT images and analysis results of bone regeneration. (A) Micro-CT images (cell-free, cell-laden and sham groups.) (B) Bone surface/volume ratio (1/mm), (C) trabecular thickness (mm), (D) connectivity density (mm^–3^), (E) bone volume (mm^3^), (F) percent bone volume (%) (BV/TV) calculated from the microCT analysis results. (*p < 0.05, **p < 0.01, ***p < 0.001, ****<0.0001).

Quantitative analysis of the microCT scans provided in Figures 4 **B–F** revealed that, in situ bioprinting of cell-laden bioink had the highest bone volume and percent bone volume, followed by the cell-free bioink, with the sham demonstrating the least. The bone surface/volume ratio and bone surface density were higher in both the cell-laden and cell-free bioink groups compared to the sham, indicating better bone formation and coverage. Structural evaluations showed the structure model index and trabecular thickness were highest in the cell-laden group, while in situ bioprinting of the cell-free bioink exhibited more favorable trabecular separation and connectivity density compared to the sham, further confirming the potential of in situ bioprinting of osteogenic bioink for bone regeneration.

### 3.3 Histopathology

The histopathological analysis of the in situ bioprinted craniofacial bone defects revealed significant differences between the groups. In defects bioprinted with cell-free bioink, a thin capsule structure was observed around the material, primarily consisting of fibrocytes (Figure 5 **A, arrowheads**). Mononuclear cell infiltrations, predominantly lymphocytes, were noted on and around the capsule, accompanied by the formation of a few new blood vessels (Figure 5A). Additionally, periosteal proliferations were seen extending into and around the material, indicating a response to the implanted scaffold (Figure 5 **A,B**).

**Figure 5:**
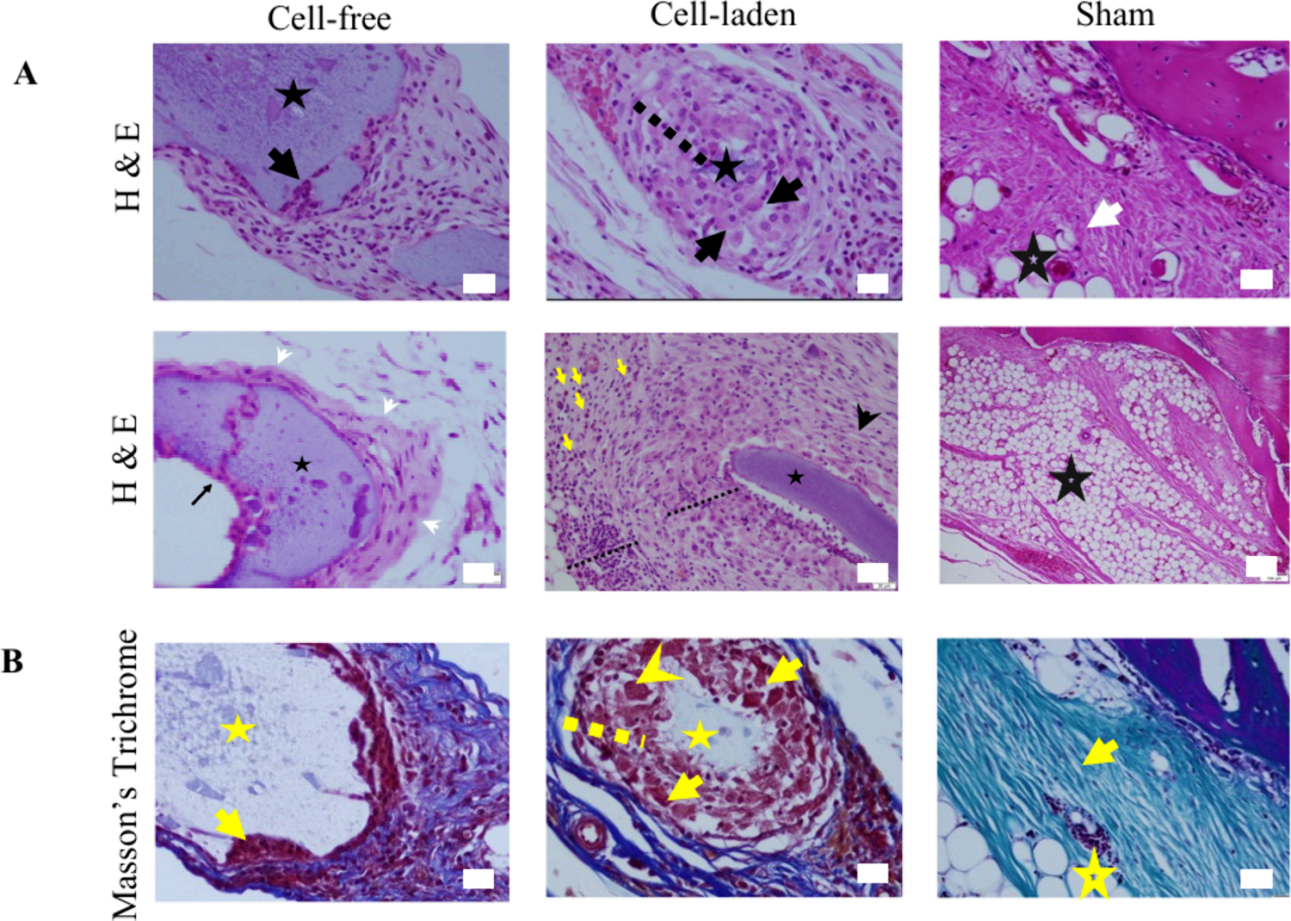
Histopathological analysis of craniofacial defect samples in longitudinal sections. (A) H&E staining. Cell-free; acellular material (black star), periosteal proliferations (arrow), and connective tissue. Cell-laden; cellular material (black star), mononuclear cell infiltration (dashed line), eccentric nucleated cell (osteoblast?) (black arrow), thin connective tissue (arrowhead), eosinophils (yellow arrows). Sham: adipose tissue (empty black star) and connective tissue (white arrow) filling the empty defect (scale bar: 20 μm). (B) Masson’s Trichrome staining. Cell-free; acellular material (star), periosteal proliferations (arrow), cell-laden; cellular material (star), mononuclear cell infiltration (dashed line), eccentric nucleated cells (osteoblasts?) (arrows), multinucleated cell (arrowhead). Sham; adipose tissue (empty yellow star) and connective tissue (yellow arrow) filling the empty defect (scale bar: 20 μm).

In contrast, defects bioprinted with cell-laden material exhibited a thicker capsule structure, primarily composed of fibroblasts and collagen fibers (Figure 5 **A, arrowhead**). This group showed numerous segmented and/or pyknotic neutrophils on the capsule and severe inflammatory cell infiltration immediately outside the capsule, consisting of lymphocytes, plasma cells, and a few macrophages (Figure 5 **A, dashed line**). The presence of many newly formed blood vessels was also noted. Large, vesicular, eccentric round nuclei with broad eosinophilic cytoplasmic cells, likely osteoblasts, were found near the material, suggesting active bone formation (Figure 5 **A,B**). A wide ring of mononuclear cell infiltrations and multinucleated cells was observed around one of the cellular materials, indicating a robust inflammatory and regenerative response (Figure 5B).

In the empty defects (sham), adipose tissue and thick trabeculae formed by connective tissue, primarily fibrocytes and collagen fibers, were observed, suggesting an attempt at repair rather than true bone regeneration (Figure 5 **A,B**). The adipose tissue and connective tissue increase in these defects likely aim at stabilizing the area, potentially forming scar tissue in the long term.

### 3.4 Immunohistochemical Analysis for Bone Regeneration and Neovascularization

Immunofluorescence staining for osteopontin (OPN) and CD31 was conducted to identify vascular structures at the defect site. OPN positivity in the cell-laden samples, along with the presence of large cells observed in histopathological staining and multinucleated cells, indicates osteoblastic differentiation of the precursor cells infiltrating the defect (Figure 6). The intensity of CD31 staining was notably higher in the cell-laden groups. Lumen formation was observed in both the cell-free and cell-laden groups (Figure 6). Among all samples, the cell-laden one exhibited the strongest CD31 staining intensity compared to the cell-free and sham groups.

**Figure 6:**
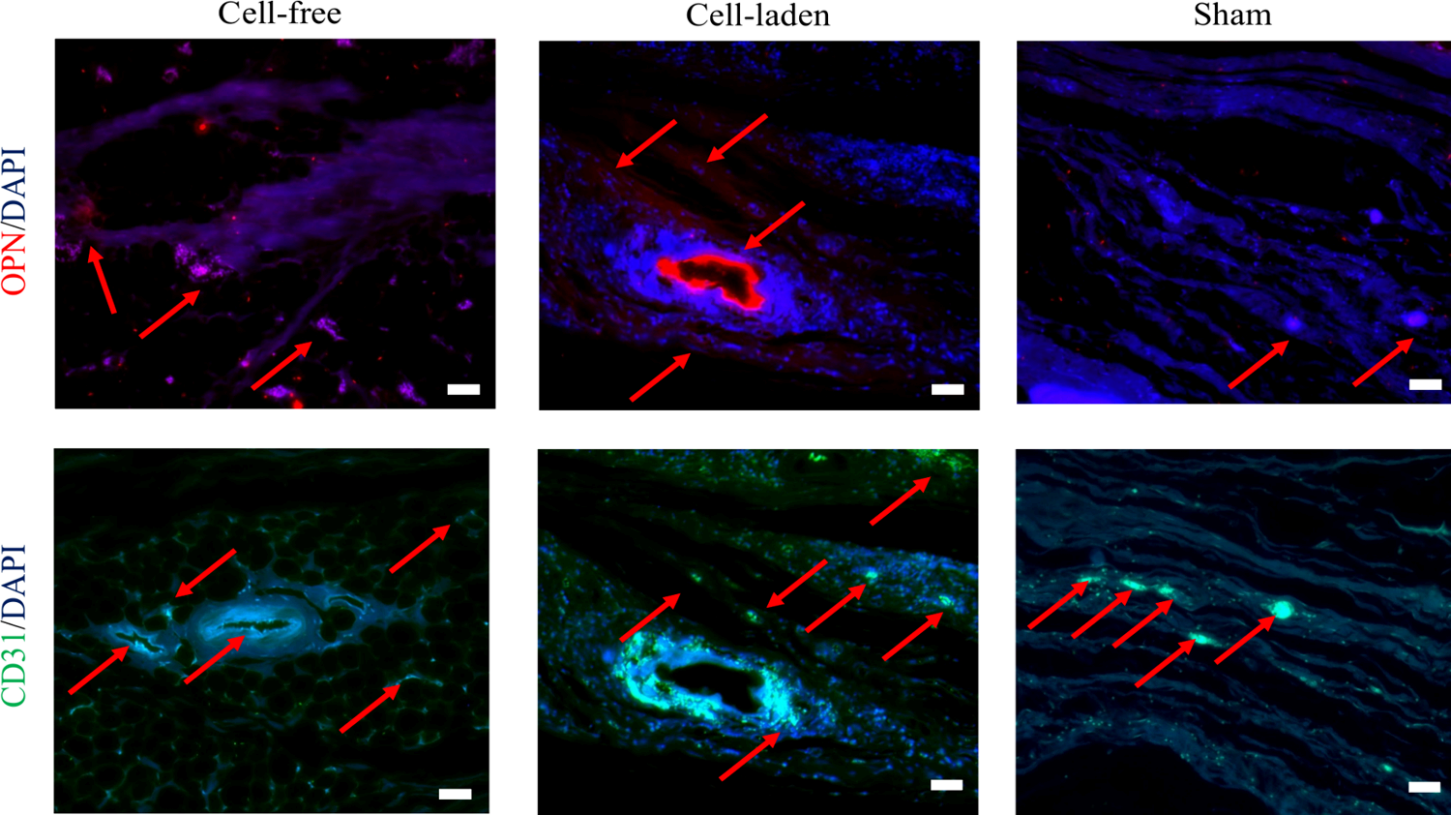
Immunohistochemical analysis of the bone regeneration and the neovascularization at the defect sites stained for osteopontin (OPN) (red), CD31 (green), and DAPI (blue) (scale bar: 50 μm).

## 4 Discussion

This study explores the potential of in situ bioprinting for craniofacial bone regeneration in a live rabbit model. By bioprinting both cell-free and cell-laden bioink onto the defect directly under anesthesia, we aimed to determine the extent to which in situ bioprinted constructs could enhance bone repair as compared to the untreated defects. Our findings provide insights into the potential clinical application of in situ bioprinting, by using a regular 3D bioprinter device for the in situ bioprinting of osteoconductive bioink formulation. This study provides an important cornerstone for the clinical relevance of in situ bioprinting for craniofacial defect repair, without the use of hand-held bioprinters that lack the positional accuracy, and complex robotic arm-based equipment which require sophisticated infrastructure.

### 4.1 Optimization of the In Situ Bioprinting Procedure

Alginate is widely used as bioink due to its favorable rheological properties, ease of gelation and stability.^33^ However, it lacks cell-binding domains and intrinsic osteoconductivity. Additionally, increasing its concentration to increase the stability and stiffness results in a nonporous network that impedes cellular infiltration and activities. To overcome these limitations, HAp, a calcium phosphate mineral naturally found in bone, was incorporated into the alginate bioink in several studies to enhance its mechanical properties and osteoconductivity.^34^

6% (w/v) alginate was used for bioprinting in this study, balancing printability and structural integrity while maintaining a suitable microenvironment for cell viability (Figure 3), since higher alginate concentrations would result in increased matrix stiffness, which can hinder cell proliferation and function by restricting nutrient diffusion and limiting cell spreading.^35,36^ Moreover, HAp was incorporated into the alginate matrix to prepare an osteoconductive bioink, leveraging its well-established role in promoting osteogenesis and mimicking the mineral composition of native bone tissue.^25,37^ The concentration of HAp was optimized to achieve proper printability, ensuring homogeneous dispersion within the alginate network. It was observed that, although printable, HAp content more than 8% w/v led to compromised extrusion (Figure 3 **A-C**). Moreover, as the HAp content increases, the mechanical strength of the material decreases due to the disruption of structural integrity caused by clumping of HAp particles. While an increase in HAp up to 8% enhances the mechanical properties, higher concentrations (16% and 24%) lead to a decline in both YounG′s modulus and ultimate compressive strength (Figure 3E). This reduction is likely due to the inability of the polymer matrix to maintain a uniform and well-integrated structure, resulting in weakened cross-linking and increased brittleness. Additionally, the 6% Alg, 4% HAp composition exhibits the lowest position in the stress–strain graph, which can be attributed to its low precross-linking viscosity (Figure 3F). The lower viscosity leads to a more loosely connected and flexible network, allowing for greater deformation under compressive loading. These findings indicate that while moderate HAp concentrations enhance mechanical performance, excessive HAp disrupts the composite structure, reducing its overall strength and stiffness.

The transition of in situ 3D printing from ex vivo and cadaver models to live animal models represented a significant advancement in validating the effectiveness of the optimized printing strategy within a biological context. It was observed on both 3D printed skull model, cadaver and the live animal under anesthesia that the bioprinted constructs led to complete coverage of the defects in line with the 3D model created from surface scanning of the craniofacial region with the defect (Figure 2 **A-C**).

### 4.2 Bone Regeneration Following In Situ Bioprinting under Anesthesia

In comparison to traditional bone grafting techniques, bioprinted constructs offer several advantages, including precise spatial control over cell and material deposition, reduced surgical invasiveness, and the ability to customize scaffold architecture in real-time. While autologous bone grafts remain the clinical gold standard, studies have shown limitations such as donor site morbidity and unpredictable resorption rates. Synthetic hydrogel-based scaffolds, like the Alg-HAp system used in this study, have been widely explored due to their biocompatibility and osteoconductive properties.^38^ demonstrated that Alg-HAp composites provide a favorable microenvironment for osteogenesis, which is consistent with our findings showing enhanced trabecular thickness and connectivity density in Alg-HAp bioprinted defects.

Notably, our study revealed that increasing HAp content beyond 8% led to a decline in mechanical strength, a trend similarly reported in prior literature. Excessive HAp loading has been shown to disrupt polymer network integrity, leading to brittle mechanical properties and suboptimal cell-matrix interactions.^39^ This highlights the importance of optimizing biomaterial composition to balance mechanical stability and biological performance.

Micro-CT analysis was performed to assess bone regeneration in craniofacial defects treated with in situ bioprinted bioinks. The significant increase in bone volume and structural integrity observed in the cell-laden bioink group aligns with previous studies highlighting the role of bioprinted cells in enhancing osteogenesis (Figure 4), which applies directly to the in situ bioprinting strategy as well. For instance,^22^ demonstrated that bioprinted cell-laden constructs promoted greater tissue integration and mineralization, reinforcing the importance of cellular contribution in biomaterial-based bone repair strategies.

Additionally, the bone surface/volume ratio and trabecular parameters in the cell-laden group were significantly improved compared to the sham and cell-free groups. These findings are in line with previous reports indicating that cell-laden constructs facilitate the secretion of osteogenic factors, enhancing matrix deposition and mineralization. Studies by^40^ further support this, showing that bioprinted stem cells within hydrogel matrices significantly enhance osteogenesis via paracrine signaling and direct differentiation. The observed increase in trabecular thickness and connectivity density in the cell-laden group further supports the hypothesis that bioprinted osteoprogenitor cells contribute to matrix deposition and mineralization, as described in other tissue engineering studies.^41^

The histopathological findings highlight the different responses elicited by cell-free and cell-laden scaffolds (Figure 5). The collagen-dominant capsule formation in the group with cell-laden material indicates a delayed fibrosis (scar formation). The increased inflammation and neovascularization in the group with cell-laden material compared to the group with cell-free material suggest a prolonged inflammatory period, allowing for more effective tissue repair before fibrosis occurs. Healing was more pronounced in defects filled with both types of materials compared to empty defects, indicating the potential of these scaffolds for effective bone regeneration. However, the empty defects were predominantly filled with adipose tissue, accompanied by significant connective tissue increase, which might form scar tissue in the long term.

The immunofluorescence staining results provided additional confirmation of the osteogenic and angiogenic responses at the defect site (Figure 6). The significant presence of osteopontin (OPN)-positive cells in the cell-laden bioink group suggests active osteoblastic differentiation, a finding consistent with previous work showing OPN as a marker of early osteogenesis.^42^ The multinucleated cells observed in histopathological staining further support the hypothesis that precursor cells differentiate into osteoblast-like cells, contributing to new bone formation. Similarly, the increased CD31 staining intensity in the cell-laden group suggests a higher degree of vascularization, which is essential for bone tissue remodeling and regeneration. Studies have shown that enhanced vascularization in biomaterial-based bone grafts can significantly improve bone healing by providing necessary oxygen and nutrients.^43^

Therefore, this study demonstrates that in situ bioprinting of a cell-laden Alg/HAp bioink significantly enhances bone regeneration in craniofacial defects. The combination of osteogenic differentiation and neovascularization observed in our results underscores the potential of this approach for future clinical applications in bone tissue engineering.

This work represents a preliminary in vivo investigation; therefore, several limitations should be acknowledged. First, while the results demonstrated promising bone regeneration, the study was conducted in an animal model where the defect was regular and with a relatively flat surface. Therefore, translation to larger animals, or humans, and on more complex irregular defects may involve additional complexities. Second, although detailed imaging and histological evaluations were performed, biomechanical testing of the regenerated bone tissue was not included, which limits direct conclusions regarding the functional strength of the new bone. Lastly, the follow-up period was limited to 6 weeks, which, while sufficient to observe early bone formation, does not provide insight into long-term remodeling or scaffold degradation. These limitations will be addressed in future studies with extended time points, larger models, and additional mechanical analyses.

## 5 Conclusions

This study presents a promising advancement in the use of in situ 3D bioprinting for craniofacial bone regeneration, demonstrating significant potential to overcome the limitations of traditional bone repair methods. By utilizing alginate and HAp bioinks and directly bioprinting onto live rabbit models, this approach achieved precise scaffold placement, full defect coverage, and successful bone integration. The results suggest that in situ bioprinting offers a rapid, patient-specific treatment strategy that can enhance bone healing by addressing key challenges such as the need for multiple surgeries and donor site morbidity. This work not only establishes a clinically relevant model for in situ bioprinting, but also provides a foundation for future research aimed at optimizing bioprinting techniques and advancing toward clinical translation. The potential for broader application in tissue engineering and regenerative medicine underscores the significance of this approach in addressing complex bone defects.

## Supplementary Material



## Notes

Rheological characterization of the bioink (PDF)
