# Supplementary material for: In Situ Bioprinting Enhances Bone Regeneration in a Live Animal Model with Craniofacial Defect
Source: ACS Biomater Sci Eng. 2025 Jul 24;11(8):5027–37. doi: 10.1021/acsbiomaterials.5c00780 (PMC12344647; doi:10.1021/acsbiomaterials.5c00780)
Supplement: Supplementary file 1 [file ab5c00780_si_001.pdf]

Supporting Information for Publication:

in situ Bioprinting Enhances Bone Regeneration in a Live Animal Model with Craniofacial Defect

Osama Ali Hindy[1], Begum Pinarbasi[1], Merve Bakici[2], Oya Burcin Demirtas[3], Seyda Gokyer[1], Arda Buyuksungur[4], Kaan Orhan[5,6], Cagdas Oto[6,7], Pinar Yilgor[1,6*]

1 Ankara University, Department of Biomedical Engineering, 06100 Ankara Turkiye

2 Kirikkale University Faculty of Veterinary Medicine Department of Surgery, 71450 Kirikkale Turkiye

3 Ankara University Faculty of Veterinary Medicine Department of Pathology, 06100 Ankara Turkiye

4 Ankara University Faculty of Dentistry, Department of Basic Medical Sciences, 06100 Ankara Turkiye

5 Ankara University Faculty of Dentistry, Department of Dentomaxillofacial Radiology, 06100 Ankara Turkiye

6 Ankara University Medical Design Research and Application Center MEDITAM, 06100 Ankara Turkiye

7 Ankara University Faculty of Veterinary Medicine Department of Anatomy, 06100 Ankara Turkiye

***Correspondence to:** Prof. Pinar Yilgor Huri (E-mail: phuri@ankara.edu.tr)

Supporting Information for Publication:

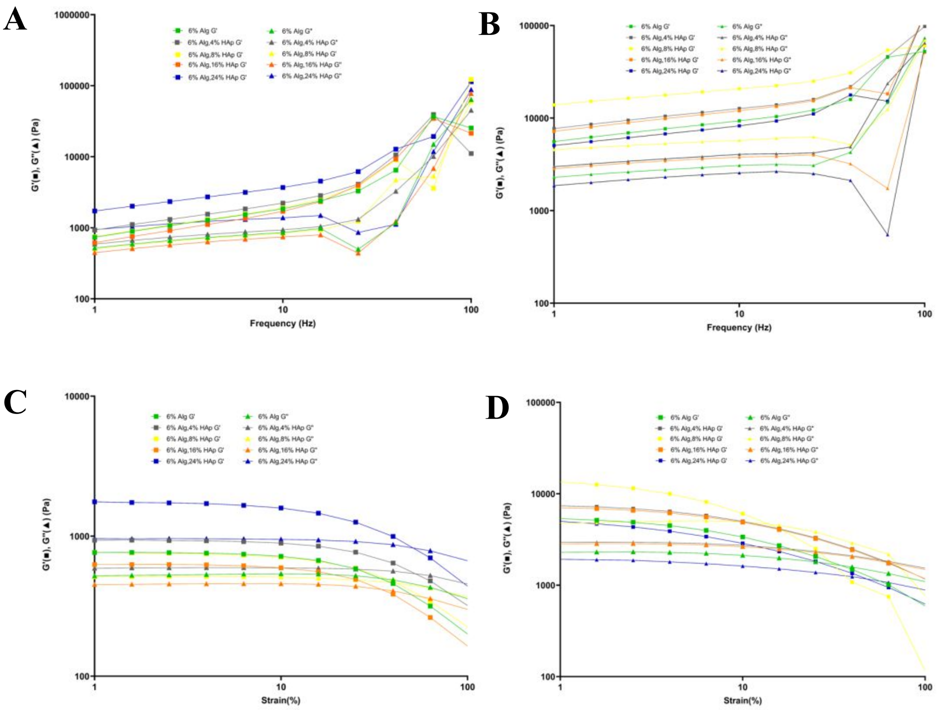

Figure S1. Rheological characterization of the bioink. **A)** Frequency sweep test with respect to angular frequency of Alg and Alg/HAp bioink with varied HAp content (4-24% (w/v) HAp) in non-crosslinked, and **B)** ionically crosslinked forms. **C)** Amplitude sweep test with respect to strain amplitude of Alg and Alg/HAp bioink with varied HAp content (4- 24% (w/v) HAp) in non-crosslinked, and **D)** ionically crosslinked forms.
